# Did the reform of the public hospitals' pay system increase the physicians' pay in China? A cross-sectional study

**DOI:** 10.3389/fpubh.2025.1555819

**Published:** 2025-06-10

**Authors:** Yaxin Zhao, Zhongliang Zhou, Xiaohui Zhai, Guanping Liu, Zhichao Wang, Qiwei Deng

**Affiliations:** ^1^College of Humanities and Social Development, Northwest A&F University, Xianyang, Shaanxi, China; ^2^School of Public Policy and Administration, Xi'an Jiaotong University, Xi'an, Shaanxi, China; ^3^School of Public Health, Health Science Center, Xi'an Jiaotong University, Xi'an, Shaanxi, China

**Keywords:** the reform of the public hospitals' pay system, physicians, total annual pay, performance-based pay, allowance

## Abstract

**Background:**

In 2017, the government initiated a pilot program for the public hospitals' pay system (RPHPS) aimed at enhancing physician compensation. We investigated the extent to which the reform impacted the physicians' pay and analyzed the factors that determine their pay.

**Methods:**

This cross-sectional study utilized China Public Hospitals' Compensation Reform Survey from 2017, which included a sample of 178,622 physicians. Total annual pay and annual performance-based pay were considered as the primary outcomes, while basic pay, allowance, and other forms of pay were classified as the secondary outcomes. We employed coarsened exact matching and hierarchical linear analysis to investigate the relationship between the RPHPS and pay, as well as the factors influencing pay.

**Results:**

The total annual pay and annual performance-based pay of physicians in the exposure group were significantly higher by 6.3% (coef: 0.06; *p* < 0.01) and 19.2% (coef: 0.19; *p* < 0.001), respectively, compared to the control group. We did not find a significant relationship between RPHPS and basic pay or allowances. Physicians' pay was associated with gender, age, educational status, professional titles, years of working, and departments. Male physicians received 4% higher total annual salary and 6% higher performance-based pay than their female counterparts.

**Conclusion:**

Positive relationships were observed between the RPHPS and both the total annual pay and performance-based pay of physicians. Significant gender disparities were identified in total annual pay, performance-based pay, and allowances. The government should promote the RPHPS to enhance physicians' pay and implement initiatives aimed at achieving equal pay for equal work, irrespective of gender.

## Introduction

The health workforce is a fundamental pillar of the healthcare system (1). Several studies have demonstrated that low educational levels, inadequate pay, and insufficient benefits are among the most pressing issues faced by physicians (2). Determining the optimal compensation structure for physicians and establishing an appropriate pay level remain long-standing dilemmas (3). The physician compensation system in public hospitals serves as a policy lever to motivate physicians, modify their excessive medical treatment behavior (4, 5), and improve the quality of medical care (6).

In many European countries, pay negotiations for physicians are conducted between the state-level governments and employee unions, with physicians' pay managed on a national scale (7). In England, general practitioners (GPs) receive a combination of capitation, one-off allowances, service items, and quality incentives (8, 9). In the United States, physicians are compensated through three primary models: fee-for-service (FFS), capitation, and salary-only structured compensation schemes. FFS, the predominant physician payment model, has been beneficial for many medical specialties but has contributed to the ongoing decline in the primary care workforce. Capitation offers financial flexibility but presents challenges related to risk adjustment. The salary-only structured compensation scheme provides a fixed amount paid without incentive plans (10). In Canada, FFS remains the primary physician payment model (11). In low- and middle-income countries (LMICs), blended payment models (e.g., fee-for-service, case-based, and capitation) were introduced to incentive efficiency (12). In Brazil, a mixed case-based and FFS payment system has been widely used. Moreover, variable compensation approximately 10% of the base wage of healthcare professional is employed to mobilize them (13). In Vietnam, the hospital autonomy reform allowed revenues to be used for paying performance-based salary with no restricting cap on this fund (14).

The salaries of physicians in developed countries are higher than those in LMICs. In the United States, it was reported that the average pay for physicians at 24 public medical schools was $240,173 in 2015 (15). In contrast, a study conducted in Georgia reported that 50% of tuberculosis physicians earned a monthly pay of only USD 205 (16). However, the average annual salary for junior doctors in Poland in 2015 ranged from USD 10,270 to USD 12,603 (17). Low wages are frequently identified as a primary factor driving the migration of physicians in LMICs (18). Therefore, it is imperative for governments to incentivize physicians by revising pay scales and raising salaries (19).

In China, physicians at public hospitals are typically compensated through a post-performance pay mechanism. Their total annual remuneration primarily consists of post-wage, pay scale wage, performance-based pay, allowances, and other pay. Basic pay is determined by factors such as job position, level, qualifications, and years of experience. Performance-based pay is contingent upon the profits generated by hospitals and their respective departments. The salary standards for public hospitals are strictly regulated by the government and must remain within a predetermined ceiling. Research indicates that physicians' pay in China is relatively low, failing to reflect the labor value of medical professionals (20–22). Furthermore, reports have highlighted a persistent shortage of GPs and pediatricians, with nearly half of all doctors leaving their positions due to inadequate compensation (23). In 2020, GPs in China earned an annual salary ranging from $1,449.30 to $2,173.95. Therefore, it is crucial to consider an appropriate increase in physician salaries.

To motivate physicians and enhance public interest in public hospitals, the Chinese government proposed the “Two Allowances” policy in August 2016. First, public hospitals are permitted to exceed the wage-control levels established for public institutions by the government, a flexibility that was not available prior to the reform. Second, public hospitals are allowed to utilize medical surpluses to reward employees after allocating various funds, which was also not permitted before the reform. This policy effectively raises salary standard and broadens the scope of performance-based pay for physicians. In January 2017, the Chinese government issued guidelines for the pilot reform of the public hospitals' pay system (RPHPS). The government selected three cities in each from the 11 pilot provinces undergoing comprehensive reform of public hospitals, as well as one city from each of the remaining provinces, excluding Tibet, to implement RPHPS in 2017. This selection constitutes the first batch of pilot cities and hospitals. In December 2017, the Chinese government issued guidelines for the expansion of the RPHPS. The guidelines mandated that cities, other than those in the initial batch of pilot cities, select at least one public hospital to implement RPHPS in 2018. This selection constitutes the expanded of pilot cities and hospitals. Numerous provinces initiated the reform, which includes measures such as exploring a target annual pay system, gradually increasing the physicians' pay, and optimizing the pay structure.

A growing number of studies have focused on physicians' pay and the factors that influence it, as well as the gender disparities in physicians' pay (15, 20, 24, 25). Despite the significance of the RPHPS in China, there is limited understanding of whether and to what extent the PHCSR affects the physicians' pay. Existing research on the effect of RPHPS has been constrained by self-reported questionnaires, a lack of administrative data, small sample sizes, and geographical limitations confined to a single province. Moreover, researchers frequently employ single-level models for data analysis, which fail to account for the hierarchical structures present in the data. For instance, physicians are often nested within hospitals, and hospitals are nested within provinces (26). To address this knowledge gap, we conducted a comparative study between districts that have implemented the policy and those that have not, examining factors that could influence physicians' pay using a hierarchical linear regression model in China.

## Methods

### Study design and setting

The study was a nationwide, cross-sectional analysis. We compared the pay of the first batch of pilot groups that implemented reform in 2017 with that of the expanded pilot groups that did not implement reforms in the same year. The first batch of pilot hospitals served as the exposure group, while the expanded pilot hospitals were designated as the control group. After matching, the final exposure group consisted of 257 hospitals, whereas the control group included 144 hospitals. The exposure group adopted the “Two Allowances” policy, reformulated the total pay standards, implemented a target annual pay system, and was allowed to utilize the surplus medical income and expenditures to reward staff. In contrast, no such changes were made in the control group.

### Data source and study sample

**Figure 1:**
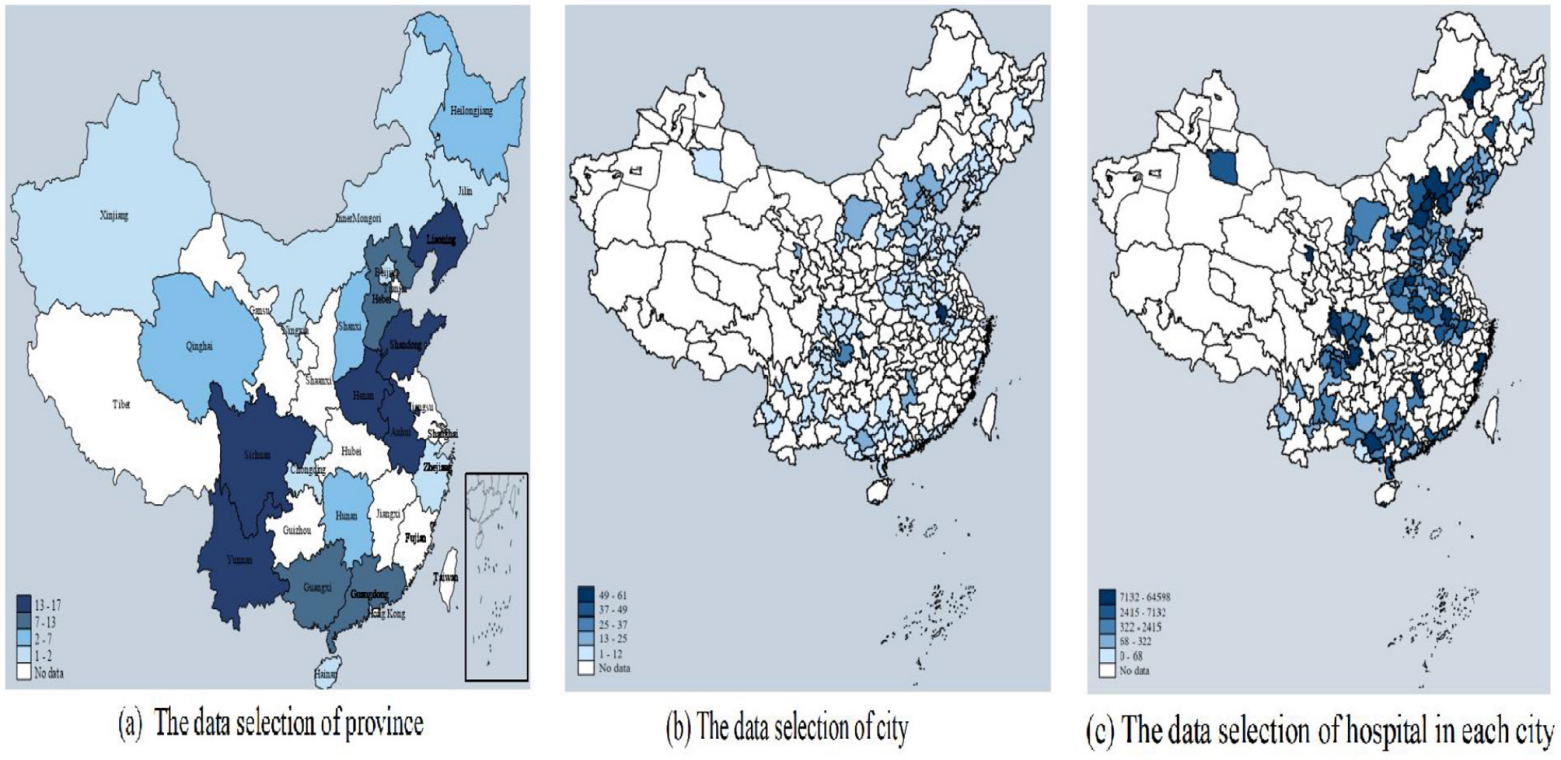
Graphical illustration of data selection. This figure means the distribution of the sample. **(a)** Shows the province distribution of the data; **(b)** shows the city distribution of the data; **(c)** shows the hospital distribution in each city of the data.

**Figure 2:**
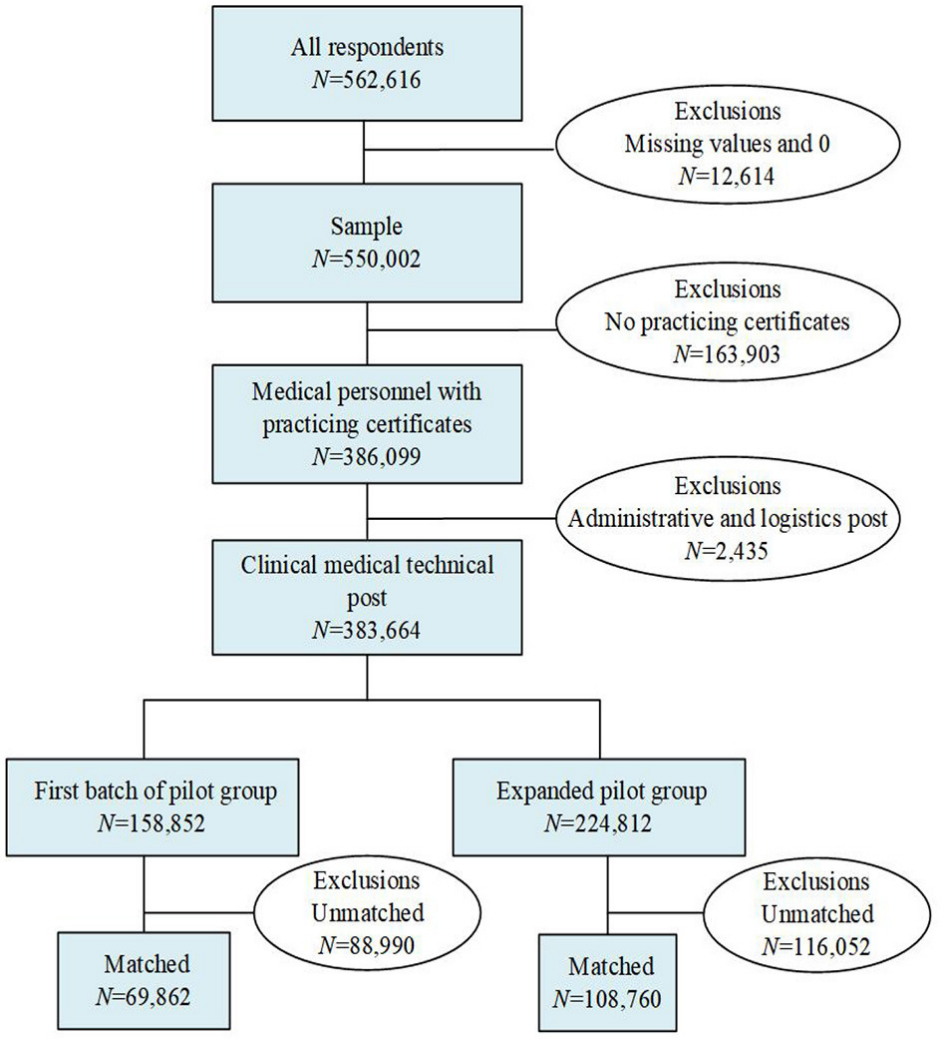
Process of sample cleaning.

The data were derived from China Public Hospitals' Compensation Reform Survey, conducted by the Department of Personnel of the Health Commission (DPHC) and the Health Development Research Centre in June 2018. This survey collected information on physicians from 1 January 2017 to 31 December 2017, which was extracted from the hospital database from the finance department of each hospital. The DPHC collected the information of physicians from initial and expanded batches of pilot hospitals. Last, the sample included 21 provinces, 188 cities, 699 hospitals, and 562,616 health workers. The sample did not consist of every public hospital in each province and accounted for approximately 12% of 21 provinces. Figure 1 shows the graphical illustration of data selection. We chose physicians who had obtained practicing certificates and who were working in the clinical medical technical post as the study sample. The final sample included 178,622 physicians after matching (see Figure 2). The data included hospital-level information (hospital type, hospital level) and physician-level characteristics (physician's gender, age, education status, work characteristics, position, departments, total annual pay, and different types of pay). In addition, the provincial-level data (gross domestic product (GDP) per capita and average social wages of all workers) were collected from the 2018 China statistical yearbook.

### Variables

The primary focus of the evaluation of the RPHPS was pay. Therefore, we used the total annual pay and annual performance-based pay in CNY as the primary outcomes, while post-wage, pay scale wage, allowances, and other forms of salary were considered secondary outcomes. Pay was derived exclusively from medical activities.

The primary independent variable was the group, which indicated whether or not the RPHPS had been implemented. We selected control variables at the province, hospital, and individual levels. Specifically, we controlled for gross domestic product per capita (GDP) and the average pay of urban workers, hospital type, hospital level, gender, age, educational status, work characteristics, position, professional titles, and departments (see Table 1).

**Table 1:** Variable definitions.

**Variables**	**Type of variables**	**Description of variables**
**Independent variables**		
Group	Binary variable	0 = Control group, reference; 1 = Treatment group
**Provincial-level**		
GDP per capita (10,000 CNY)	Continuous variable	Continuous variable
Average pay of the urban worker (10,000 CNY)	Continuous variable	Continuous variable
**Hospital-level**		
Hospital level	Categorical variable	0 = Ungraded hospitals, reference; 1 = Secondary hospitals; 2 = Tertiary hospitals
Hospital type	Categorical variable	0 = General hospital, reference; 1=Traditional Chinese medicine hospital; 2 = Specialized hospital
**Individual-level**		
Gender	Binary variable	0 = Female, reference, 1 = Male
Age (years)	Continuous variable	Continuous variable
Education status	Categorical variable	0 = Secondary vocational diploma/ Junior High School, reference; 1 = vocational diploma; 2 = bachelor; 3 = Masters and above
Work characteristics	Categorical variable	0 = Non-establishment staff, reference; 1 = Establishment staff
Position	Categorical variable	0 = General staff, reference; 1 = Section leader; 2 = Hospital manager
Professional titles	Categorical variable	0 = No title, reference; 1= Primary professional title; 2 = Intermediate title; 3 = Associate senior title; 4 = Senior title
Years of experience	Continuous variable	Continuous variable
Departments	Categorical variable	0 = Clinical departments, reference; 1 = Medical detection departments; 2 = Logistics departments; 3 = Administrative departments; 4 = Other departments
**Dependent variables**		
Total annual salary (CNY)	Continuous variable	Continuous variable
Performance-based pay (CNY)	Continuous variable	Continuous variable
Post-wage (CNY)	Continuous variable	Continuous variable
Pay scale wage (CNY)	Continuous variable	Continuous variable
Allowance (CNY)	Continuous variable	Continuous variable
Other pay (CNY)	Continuous variable	Continuous variable

### Statistical analysis

#### Coarsened exact matching

Coarsened exact matching (CEM) is a non-parametric matching technique designed to reduce imbalance between treatment and control groups in observational studies. CEM matches units based on coarsened values of covariates, ensuring that treatment and control groups are balanced across these variables. This method is particularly effective in reducing model dependence and bias caused by covariate imbalance. We employed the CEM method together with parametric analysis, a novel technique designed to ensure better balance in covariates between the treatment and control group, thereby enhancing the robustness of the analysis (27). The multivariate imbalance measure *L1* was used to assess balance in the multivariate distribution of covariates between the initial and expanded groups. It is calculated as the sum of absolute differences in the empirical distribution functions of the covariates across groups. The values of *L1* range from 0 to 1, where 0 indicates that the data from the two comparison groups are completely balanced, and 1 signifies complete imbalance (28). A substantial reduction in imbalance across the coarsening variables, with *L1* approaching zero after matching, indicates the matching performance (29). The basic algorithm of CEM consists of three procedures. First, all covariates (GDP per capita, average pay of the urban worker, hospital level, hospital type, gender, age, work characteristics, education status, professional titles, position, years of experience, departments) are coarsened by recoding them into groups. In the second step, the exact matching algorithm is employed to categorize physicians into strata based on these coarsened variables. Finally, the matched data are retained, while the unmatched data are discarded. A weight variable is generated by the CEM method to equalize the number of subjects in the two groups within each layer.

#### Hierarchical linear model

The hierarchical linear model (HLM) is the preferred method for analyzing hierarchical data (26). Given that the dependence among samples arises from multiple levels—such as physicians being grouped within hospitals and hospitals being grouped within provinces—we employed HLM to estimate both micro- and macro-effects (26, 30). Before applying this method, we utilized the intraclass correlation coefficient (ICC) to assess the validity of our HLM. The ICC measures the degree of correlation among observations within a cluster and ranges from 0 to 1. If the ICC varies significantly across multiple levels, then HLM is deemed appropriate for the analysis. Specifically, the degree of within-group correlation is considered small if the ICC > 0.01, medium if the ICC > 0.1, and large if the ICC > 0.25. A higher ICC value indicates a greater influence of group membership on individual ratings (31). The ICC is calculated using Equation 1 (32):


(1) $$\begin{array}{l} \text{ICC}=\frac{\sigma_{\mu 0}^{2}}{\sigma_{\mu 0}^{2}+\sigma_{e0}^{2}} \end{array}$$

where $\sigma_{\mu 0}^{2}$ is the between-group variance, and $\sigma_{e0}^{2}$ is the variance of the observations.

Due to the skewed distribution of pay, HLM was conducted using the natural logarithms of the outcome. The three levels of the multiple linear regression model were calculated using


(2) $$\begin{array}{l} y_{ijk}=\beta x_{ijk}+\gamma \omega_{jk}+\eta z_{k}+\varepsilon_{ijk}+\mu_{jk}+\nu_{k} \end{array}$$

where *y*_*ijk*_ is the continuous outcome; i, j, and k represent the physicians-level, hospital-level and provincial-level, respectively; *x*_*ijk*_, ω_*jk*_, and *z*_*k*_ represent the independent variables of medical-personnel-level 1, hospital-level 2, and provincial-level 3, respectively; β, γ, and η represent the fixed regression coefficients of the explanatory variables at level 1, level 2, and level 3, respectively; ε_*ijk*_, μ_*jk*_, and ν_*k*_ are multilevel residuals.

All analyses were performed using Stata 14.0 (StataCorp, College Station, TX, USA). To characterize the matching results, we compared them using Student's *t*-test for continuous variables and a chi-squared test for categorical variables and the sample after matching using the CEM weights. All significance levels were set at a *p* < 0.05.

## Results

### Coarsened exact matching

The values of *L1* and mean statistics are displayed in Table 2. Matching revealed that the *L1* values for the samples and all variables between the two groups were close to zero and much lower than before. This shows that the match results between the groups were favorable, and the two groups became more comparable. The CEM was successful in balancing the covariates between the two groups.

**Table 2:** L1 measure of imbalance before and after coarsened exact matching.

**Coarsening variables**	**Before matching**	**After matching**
	***L*** **1 (mean)**	***L*** **1 (mean)**
ln (GDP per capita)	0.580 (−0.072)	0.002 (< 0.010)
ln (average pay of the urban worker)	0.653 (−0.025)	0.010 (−0.001)
Hospital level	0.062 (−0.055)	< 0.010 (< 0.010)
Hospital type	0.036 (0.024)	< 0.010 (< 0.010)
Gender	0.015 (0.015)	< 0.010 (< 0.010)
Age	0.028 (−0.048)	< 0.010 (< 0.010)
Work characteristics	0.008 (−0.008)	< 0.010 (< 0.010)
Education status	0.041 (−0.016)	< 0.010 (< 0.010)
Professional titles	0.017 (−0.060)	< 0.010 (< 0.010)
Position	0.010 (−0.009)	< 0.010 (< 0.010)
Years of experience	0.018 (−0.042)	< 0.010 (< 0.010)
Departments	0.029 (0.099)	< 0.010 (< 0.010)
Multivariate *L*1	0.799	0.010
*N*	383,664	178,622

Treated physicians (69,862 matched, 88,990 unmatched) were those who worked in piloted hospitals, whereas untreated physicians (108,760 matched, 116,052 unmatched) comprised those in other hospitals that still not pilot the reform. The parameter L1 here is pre- and post-matching covariate imbalance, which is the calculated value of L1 for the jth separate variable. The means are shown in parentheses to illustrate their differences in means.

### Descriptive analysis and summary of the outcomes

Table 3 lists the distribution of the provincial-level, hospital-level, and physician-level characteristics of the two groups before and after matching. The total sample was comprised of 69,862 physicians in the exposure group and 108,760 physicians in the control group. After matching, it was observed that the majority of physicians were from tertiary hospitals (88.5%) and general hospitals (87.56%). The majority of those were female (70.73%), establishment staff (52.29%), had a bachelor's degree (55.04%), and had a primary professional title (52.81%). The mean age of the sample was 35.13 (SD: 8.72). Moreover, the results of the matching revealed that the characteristics of the two groups were not significantly different.

**Table 3:** Descriptive statistics for control variable before and after coarsened exact matching.

**Control variables**	**Before matching**			**After matching**		
	**Control group**	**Treatment group**	***p*** **-value**	**Control group**	**Treatment group**	***p*** **-value**
**Provincial-level**						
ln (GDP per capita) (10,000 CNY)	1.64 (0.30)	1.57 (0.23)	< 0.001	1.53 (0.17)	1.53 (0.17)	0.500
ln (Average pay of the urban worker) (10,000 CNY)	1.91 (0.18)	1.89 (0.06)	< 0.001	1.88 (0.06)	1.88 (0.06)	0.238
**Hospital level**						
Unrated	1,625 (0.72)	445 (0.28)	< 0.001	246 (0.23)	158 (0.23)	0.996
Secondary hospitals	56,366 (25.07)	50,559 (31.83)		12,264 (11.28)	7,878 (11.28)	
Tertiary hospitals	166,821 (74.20)	107,848 (67.89)		96,249 (88.50)	61,826 (88.50)	
**Hospital type**						
General hospital	160,348 (71.33)	116,413 (73.28)	< 0.001	95,230 (87.56)	61,171 (87.56)	0.998
Traditional Chinese medicine hospital	27,465 (12.22)	26,129 (16.45)		6,428 (5.91)	4,129 (5.91)	
Specialized hospital	36,999 (16.46)	16,310 (10.27)		7,170 (6.53)	4,562 (6.53)	
**Individual-level**						
**Gender**						
Female	174,906 (77.97)	117,353 (75.60)	< 0.001	85,155 (78.30)	54,700 (78.30)	0.998
Male	49,416 (22.03)	37,884 (24.40)		23,604 (21.70)	15,162 (21.70)	
Age (years)	35.64 (9.23)	35.18 (9.12)	< 0.001	35.13 (8.75)	35.13 (8.72)	0.991
**Work characteristics**						
Non-establishment staff	108,327 (48.19)	76,109 (47.91)	< 0.001	51,890 (47.71)	33,332 (47.71)	1.000
Establishment staff	116,485 (51.81)	82,743 (52.09)		56,869 (52.29)	36,530 (52.29)	
**Education status** ^§^						
Secondary vocational diploma/Junior high school	18,939 (8.44)	10,961 (6.91)	< 0.001	4,782 (4.40)	3,072 (4.40)	0.999
Vocational diploma	66,300 (29.54)	51,850 (32.67)		32,154 (29.56)	20,654 (29.56)	
Bachelor	113,024 (50.36)	80,647 (50.81)		59,857 (55.04)	38,449 (55.04)	
Master and above	26,175 (11.66)	15,270 (9.62)		12,106 (11.00)	7,686 (11.00)	
**Professional titles** ^¶^						
No title	8,656 (3.93)	8,632 (5.53)	< 0.001	3,149 (2.90)	2,024 (2.90)	0.999
Primary professional title	116,073 (52.76)	81,881 (52.46)		57,292 (52.81)	36,827 (52.81)	
Intermediate title	61,512 (27.96)	43,851 (28.09)		32,785 (30.22)	21,074 (30.22)	
Associate senior title	23,895 (10.86)	16,057 (10.29)		11,411 (10.52)	7,335 (10.52)	
Senior title	9,860 (4.48)	5,669 (3.63)		3,841 (3.54)	2,469 (3.54)	
**Position**						
General staff	196,222 (87.28)	140,070 (88.27)	< 0.001	98,888 (90.92)	63,521 (90.92)	0.999
Section leader	27,202 (12.10)	17,559 (11.07)		9,626 (8.85)	6,183 (8.85)	
Hospital manager	1,388 (0.62)	1,047 (0.66)		246 (0.23)	158 (0.23)	
**Departments**						
Clinical departments	171,636 (76.35)	117,555 (74.00)	< 0.001	90,837 (83.52)	58,349 (83.52)	0.997
Medical detection departments	31,817 (14.15)	21,558 (13.57)		12,846 (11.81)	8,252 (11.81)	
General affairs/Logistics departments	1,265 (0.56)	745 (0.47)		123 (0.11)	79 (0.11)	
Administrative departments	9,637 (4.29)	6,567 (4.13)		2,402 (2.21)	1,543 (2.21)	
Other departments	10,457 (4.65)	12,427 (7.82)		2,551 (2.35)	1,639 (2.35)	
Years of experience	12.23 (10.01)	11.84 (9.93)	< 0.001	11.80 (9.71)	11.76 (9.69)	0.215
*N*	224,812	158,852		108,760	69,862	

The descriptive statistics are calculated on the pre- and post-matching sample.

Continuous variables are shown as mean (SD) and categorical variables are shown as N (%).

^§^Missing data were observed for two physicians after matching.

^¶^Missing data were observed for 415 physicians after matching.

**Figure 3:**
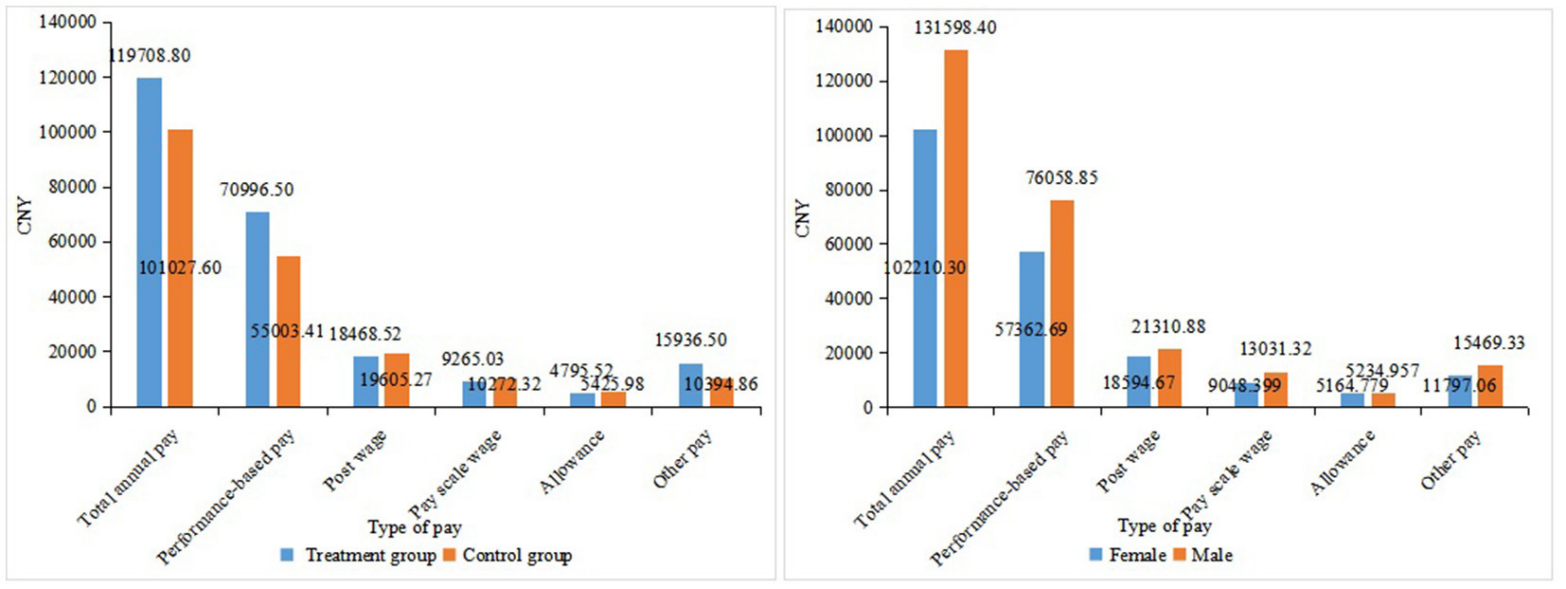
Pay by different groups and gender.

Table 4 reports the summary statistics of the outcome variables after matching. The means (SD) of total annual pay, annual performance-based pay, post-wage, pay scale wage, allowance, and other pay among physicians in 2017 were CNY 108,334.10 (55,074.39), CNY 61,258.57 (46,035.35), CNY 19,160.67 (6,315.85), CNY 9,878.35 (8,308.17), CNY 5,179.40 (9,602.9), and CNY 12,562.28 (20,090.86), respectively. Figure 3 depicts the pay by different groups and gender. Compared with the control group, the total annual pay, performance-based pay, and other pay in the exposure group were higher. However, the post-wage, pay scale wage, and allowance in the exposure group were lower than that in the control group. All the different types of pay among female physicians in 2017 were lower than male physicians.

**Table 4:** Summary statistics for total outcome variables after matching.

**Outcome variable**	**Obs**.	**Mean**	**Std. dev**.	**Min**.	**Max**.
Total annual pay (CNY)	178,622	108,334.10	55,074.39	17,832	291,842.90
Performance-based pay (CNY)	178,622	61,258.57	46,035.35	0	223,299.80
Post-wage (CNY)	178,622	19,160.67	6,315.85	1,490	38,440
Pay scale wage (CNY)	178,622	9,878.35	8,308.17	0	34,836
Allowance (CNY)	178,622	5,179.40	9,602.90	0	49,404
Other pay (CNY)	178,622	12,562.28	20,090.86	0	105,600

The summary statistics are calculated on the post-matching sample.

### Hierarchical linear model estimates

The fitted results of the three-level empty model after matching are shown in Table 5. The results showed that the ICC values were all above 0.1 at both the provincial level and the hospital levels, indicating that the HLM was appropriate for the study.

**Table 5:** Three-level empty model of factors associated with salary after matching.

**Variables**	**ln (total annual pay)**	**ln (performance- based pay)**	**ln (post-wage)**	**ln (pay scale wage)**	**ln (allowance)**	**ln (other pay)**
**Fixed effects**						
Constant	11.36 (0.05)^***^	10.36 (0.14)^***^	9.78 (0.03)^***^	8.31 (0.16)^***^	6.89 (0.41)^***^	6.08 (0.42)^***^
**Random effects**						
Level 3	0.03 (0.01)^***^	0.26 (0.12)^***^	0.01 (0.01)^***^	0.26 (0.15)^***^	2.06 (0.89)^***^	1.65 (0.88)^***^
Level 2	0.12 (0.01)^***^	0.40 (0.03)^***^	0.12 (0.01)^***^	2.30 (0.17)^***^	7.31 (0.53)^***^	17.07 (1.22)^***^
Residual	0.15 (0.01)^***^	0.26 (0.01)^***^	0.11 (0.01)^***^	5.20 (0.02)^***^	3.34 (0.01)^***^	1.39 (0.01)^***^
**ICC**						
Level 3	0.10 (0.03)^***^	0.27 (0.11)^***^	0.10 (0.02)^***^	0.10 (0.02)^***^	0.16 (0.06)^***^	0.11 (0.04)^***^
Level 2	0.50 (0.03)^***^	0.72 (0.05)^***^	0.53 (0.02)^***^	0.33 (0.02)^***^	0.73 (0.02)^***^	0.93 (0.01)^***^
Observation	178,622	178,622	178,622	178,622	178 622	178 622

The multilevel linear analysis is calculated on the post-matching sample with the CEM weights. Significance level: ^***^p < 0.001.

Table 6 shows the multilevel linear analysis after matching. The total annual pay and annual performance-based pay in the exposure group were significantly 6.3% and 19.2% higher than that in the control group (coef: 0.06, *p* < 0.01; coef: 0.19, *p* < 0.001; respectively). The estimates for post-wage, pay scale wage, allowance, and other pay are not statistically significant. A positive association was observed between the average earnings of an urban worker and the total annual pay (coef: 2.28; *p* < 0.001). We also found that the total annual pay and annual performance-based pay in specialized and traditional Chinese medicine hospitals were substantially lower than those in general hospitals. Compared to their counterparts, physicians who were older, had a vocational diploma or above, had professional titles, were section leaders, or were hospital managers earned significantly higher total annual pay and annual performance-based pay. It was found that clinical departments had significantly higher total annual pay and annual performance-based pay than logistics, administrative, and other departments. Moreover, the results showed that male physicians received 4% higher total annual pay (coef: 0.04; *p* < 0.001) and 6% higher performance-based pay (coef: 0.06; *p* < 0.01) than females, while male physicians had 65% lower allowance than female physicians (coef: −0.65; *p* < 0.001). We did not find significant gender difference in basic pay.

**Table 6:** Multilevel linear analysis of factors associated with physicians' pay after matching.

**Variables**	**ln (total annual pay)**	**ln (performance-based pay)**	**ln (post-wage)**	**ln (pay scale wage)**	**ln (allowance)**	**ln (other pay)**
**Fixed effects**						
Group	0.06 (0.03)^**^	0.19 (0.07)^***^	−0.04 (0.03)	−0.06 (0.16)	0.16 (0.48)	−0.04 (0.64)
ln (GDP per capita) (10,000 CNY)	0.22 (0.09)^**^	0.14 (0.39)	−0.12 (0.10)	−0.94 (0.31)^***^	−2.57 (1.48)	−0.53 (2.04)
ln (Average pay of the urban worker) (10,000 CNY)	2.28 (0.27)^***^	1.92 (1.24)	0.32 (0.27)	2.39 (1.08)^*^	11.86 (3.80)^***^	7.37 (4.92)
Secondary hospital	−0.27 (0.03)^***^	−0.33 (0.06)^***^	−0.01 (0.03)	−0.80 (0.53)	1.82 (0.27)^***^	−1.75 (0.47)^***^
Tertiary hospital	−0.02 (0.05)	−0.03 (0.09)	0.03 (0.05)	−0.59 (0.52)	2.35 (0.40)^***^	−0.69 (0.44)
Traditional Chinese medicine hospital	−0.19 (0.03)^***^	−0.36 (0.03)^***^	0.03 (0.04)	−0.15 (0.16)	0.23 (0.36)	−0.24 (0.31)
Specialized hospital	−0.09 (0.03)^***^	−0.09 (0.04)^**^	0.09 (0.04)^*^	−0.49 (0.53)	2.55 (0.21)^***^	−1.08 (0.43)^*^
Male	0.04 (0.01)^***^	0.06 (0.01)^**^	0.01 (0.02)	0.03 (0.03)	−0.65 (0.17)^***^	0.06 (0.03)
Age (years)	0.01 (0.01)^***^	0.01 (0.01)^***^	0.01 (0.01)	0.03 (0.01)^*^	−0.04 (0.02)^***^	0.01 (0.01)
Establishment staff	0.09 (0.03)^***^	0.05 (0.02)^***^	0.09 (0.03)^**^	1.55 (0.71)^*^	1.05 (0.62)	0.17 (0.12)
Vocational diploma	0.03 (0.01)^***^	0.05 (0.02)^***^	−0.04 (0.03)	0.26 (0.13)^*^	0.24 (0.09)^***^	0.05 (0.02)^*^
Bachelor	0.10 (0.02)^***^	0.13 (0.02)^***^	0.05 (0.04)	0.56 (0.10)^***^	0.39 (0.16)^*^	0.11 (0.02)^***^
Masters and above	0.09 (0.01)^***^	0.07 (0.03)^***^	0.12 (0.06)^*^	0.38 (0.15)^**^	0.09 (0.19)	0.08 (0.04)
Primary professional title	0.46 (0.14)^***^	0.58 (0.21)^***^	0.23 (0.08)^***^	3.50 (0.69)^***^	1.59 (0.40)^***^	0.99 (0.36)^***^
Intermediate title	0.59 (0.13)^***^	0.76 (0.21)^***^	0.31 (0.07)^***^	3.92 (0.75)^***^	1.57 (0.39)^***^	1.07 (0.37)^***^
Associate senior title	0.71 (0.14)^***^	0.88 (0.21)^***^	0.51 (0.09)^***^	3.83 (0.74)^***^	0.99 (0.27)^***^	1.1 (0.40)^***^
Senior title	0.81 (0.15)^***^	0.99 (0.22)^***^	0.69 (0.11)^***^	3.77 (0.75)^***^	0.62 (0.29)^***^	1.1 (0.43)^***^
Section leader	0.11 (0.02)^***^	0.14 (0.01)^***^	0.03 (0.01)^*^	−0.18 (0.08)^*^	0.18 (0.09)	0.12 (0.06)
Hospital manager	0.28 (0.04)^***^	0.38 (0.04)^***^	0.04 (0.04)	0.07 (0.23)	0.28 (0.21)	0.25 (0.15)
Medical detection departments	−0.01 (0.01)	−0.01 (0.02)	−0.01 (0.01)	−0.10 (0.03)^***^	−0.04 (0.03)	−0.04 (0.02)
General affairs/Logistics departments	−0.09 (0.02)^***^	−0.17 (0.03)^***^	−0.01 (0.04)	0.12 (0.20)	0.18 (0.15)	−0.06 (0.07)
Administrative departments	−0.13 (0.02)^***^	−0.21 (0.04)^***^	−0.01 (0.01)	−0.35 (0.23)	−0.13 (0.12)	−0.13 (0.05)^***^
Other departments	−0.18 (0.04)^***^	−0.31 (0.08)^***^	−0.04 (0.04)	−0.26 (0.08)^***^	−0.36 (0.08)^***^	−0.19 (0.08)^*^
Years of experience	0.01 (0.01)^***^	0.01 (0.01)	0.01 (0.01)^***^	0.04 (0.01)^***^	0.07 (0.01)	0.01 (0.01)
**Random effects**						
Level 3	0.01 (0.01)^***^	0.11 (0.06)^***^	0.01 (0.01)^***^	0.06 (0.05)^***^	1.28 (0.47)^***^	1.02 (0.56)^***^
Level 2	0.08 (0.01)^***^	0.35 (0.07)^***^	0.10 (0.04)^***^	0.17 (0.42)^***^	6.95 (1.25)^***^	16.86 (1.12)^***^
**Observation**	178,622	178,622	178,622	178,622	178,622	178,622

The multilevel linear analysis is calculated on the post-matching sample with the CEM weights. Significance level: ^*^p < 0.05; ^**^p < 0.01; ^***^p < 0.001.

## Discussion

An important contribution to our study is that, to the best of our knowledge, it is the first empirical research to examine the relationship between the RPHPS and physicians' pay. There are three interesting findings. First, the average total annual pay and performance-based pay for physicians were CNY 108,334.10 and CNY 61,258.57, respectively. Second, the implementation of RPHPS improved both total pay and performance-based pay. Moreover, the pay of physicians was influenced by hospital type, gender, age, education status, work characteristics, position, professional titles, and years of experience. Gender differences existed in total pay, performance-based pay, and allowance, but there were no significant differences in basic pay.

The average total annual pay of CNY 108,334.10 (US$ 16,033.44) for Chinese physicians in our investigation is higher than the figure reported for Chinese public tertiary hospitals in 2015 (CNY 96,414.07) (20). Our research revealed that the total annual pay was higher than those reported in developing countries. In Poland, the average annual salary of junior doctors in 2015 was between US$10,270 and US$12,603 (17). In 2011, the annual wage of the health workforce in the Russian Fed Reserve was approximately US$8,700 (33). The total annual pay we discovered in our research was less than that found in India in 2011 (US$16,241) (33). Our research found that the total annual pay was significantly lower than those reported in developed countries. In Brazil, 80% of women earn ≤ US$86,100 annually, while 51% of men earn ≥US$86,100 annually (24). In Canada, women physicians earned approximately US$268,044 in 2017 (34). The average salary for physicians in the US was approximately $250,000 in 2014 (35).

We found some interesting evidence that the implementation of RPHPS played an important role in increasing physicians' total annual pay and performance-based pay, which aligns with the aim of the RPHPS. Similar to RPHPS, Vietnam's reform empowers hospitals with autonomy over payroll, contracts, recruitment, and revenue-based income, aiming to boost performance and align pay with service quality (14). Evidence from Africa shows that physicians' pay increased between 70% and 150% after the introduction of the additional duty hour allowance scheme (36). Our results also align with research in the UK, which found that a proposed new contract payment structure lead to an increase of pay for junior doctors with the most onerous shifts (37). Existing studies have shown that raising salaries would reduce physician migration, ensure the stability of health workers' stability, and increase physicians' adherence to guidelines (17, 38). We did not find a significant relationship between RPHPS and basic pay, allowance, and other forms of pay. The differential effects of RPHPS on pay components can be explained by shifts in incentive structures and administrative discretion. The “Two Permissions” policy has granted hospitals greater autonomy in pay distribution. Hospital management can now flexibly allocate performance-based pay according to their strategic development objectives, key departmental priorities, and healthcare workers' actual performance, leading to significant growth in performance-based pay. In contrast, basic salaries remain constrained by the unified wage standards for public institutions, primarily determined by relatively fixed factors such as professional titles, positions, and years of service. These continue to follow established national and local policy standards, resulting in minimal changes.

There are several plausible explanations for our findings. In China, public hospitals are part of public institutions, and public institutions determine and provide the basic pay for physicians. The basic pay level for public institutions was relatively fixed with a small range of changes. The total amount of performance-based pay was regulated by public institutions, and it was distributed automatically by hospitals. The proportion of performance-based pay is higher than that of basic pay. Due to the current situation of small and stagnant basic pay growth, performance-based pay has become the primary method for increasing physicians' pay. The RPHPS allowed public hospitals to exceed the wage-control level of public institutions formulated by the government, which means that the total pay or performance-based pay-control level of public hospitals should be adjusted reasonably. In addition, the RPHPS permitted hospital surplus to be used primarily for personnel incentives, resulting in greater autonomy for public hospitals after RPHPS. It provides an economic incentive for the health workforce to increase their performance-based pay and total annual pay. For example, the performance-based pay-control system of public hospitals in Qinghai Province is determined by grades (A, B, C, and D). The performance-based pay of hospitals with grades A, B, or C will increase by 5%−20% of the average annual wage level of public institutions in Qinghai province. The performance-based pay-control line of public hospitals will be dynamically adjusted following the changes in regional economic level, price index, the average wage of employees, and other factors in the future.

We also found that physicians' educational levels were low, which is in line with other studies in China (39). The reason for this is that there are numerous junior medical colleges and secondary vocational schools in China, and vocational diplomas were allowed to legally enter medical professional practice without a bachelor's degree. The pay of physicians was strongly associated with the average pay of an urban worker, the hospital type, gender, age, education status, work characteristics, position, professional titles, years of experience, and departments, which is consistent with the previous studies (9, 40). We found significant gender gaps in total annual pay. A growing body of estimates has demonstrated that there are gender differences in physician pay (41). In the US public medical schools, male pay and female pay are $227,783 and $247,661, respectively (15). In Canada, hourly pay for male surgeons was 24% higher than that for female surgeons (42), and this may be because women are more likely to work less than full-time and possibly biased “merit” awards. Previous studies suggested that the pay of nurses with more than 18 years of experience was paid higher than those without experience (43). Moreover, several studies have reported that the pay of hospital management frequently exceeds that of most physicians (44).

### Strengths and limitations

This is the first study to compare the association between RPHPS and the different types of pay in a nationwide survey of Chinese physicians. Policymakers and researchers who are focused on the health workforce and payment will benefit from the study's implications. We also explored the gender gap in different types of physicians' pay, which is limited in China. The administrative data we employed were more accurate than the survey conducted using self-reported questionnaires. We used hierarchical linear analysis with coarsened exact matching to ensure better balance when comparing the pay between the exposure group and the control group. There are several limitations in the current study. First, the cross-sectional study was unable to ascertain causal interpretations between the RPHPS and physicians' pay. Second, the sample selection was determined by the piloted list selected by the governments, which was not random. Third, the absence of information on the total hospital revenue or the personnel expenditure as a proportion of total hospital expenditure could result in bias due to these omitted variables. Fourth, the “Physicians' pay Questionnaire” was created by scholars from DPHC, but it was not a valid and authoritative questionnaire worldwide. Finally, only observed variables are taken into account in matching and the potential bias of unobserved heterogeneity cannot be addressed.

## Conclusion

There is a strong relationship between the implementation of RPHPS and the increase in physicians' pay, which has policy implications for developing countries regarding how to retain physicians. From the government's perspective, there may be value in further promoting the RPHPS to encourage pay improvements. The pay of physicians was strongly associated with the average pay of an urban worker, hospital type, physicians' gender, age, education, work characteristics, position, professional titles, years of experience, and departments. There are gender disparities in the pay of physicians, except for the basic pay. Some initiatives should be taken to ensure equal pay for equal work, regardless of gender.

## Data Availability

Data from this study is not available due to their containing information that could compromise the privacy of research participants. Requests to access the datasets should be directed to the corresponding author.
