# Supplementary material for: Did the reform of the public hospitals' pay system increase the physicians' pay in China? A cross-sectional study
Source: Front Public Health. 2025 Jun 10;13:1555819. doi: 10.3389/fpubh.2025.1555819 (PMC12185482; doi:10.3389/fpubh.2025.1555819)
Supplement: Supplementary file 1 [file Data_Sheet_1.pdf]

Table S1. Summary statistics for outcome variables before matching

Outcome variable	Obs.	Mean	Std. Dev.	Min.	Max.
**Total sample**					
Total annual salary (CNY)	383,664	104,799.90	57,193.37	15,002.70	283,623.80
Annual performance-based salary (CNY)	383,664	59,714.69	47,470.47	0	223,299.80
**Treatment group**					
Total annual salary (CNY)	158,852	110,227.80	57,152.70	15,002.70	283,623.80
Annual performance-based salary (CNY)	158,852	62,448.29	48,315.35	0	223,299.80
**Control group**					
Total annual salary (CNY)	224,812	100,964.50	56,910.93	15,002.70	283,623.80
Annual performance-based salary (CNY)	224,812	57,806.12	46,777.05	0	223,299.80

Note. the summary statistics are calculated on the pre- matching sample.

Table S2. Three-level empty model of factors associated with salary before matching

	**ln (Total annual salary)**		**ln (Annual performance-based salary)**	
	**Coeff. (SD)**	**95%CI**	**Coeff. (SD)**	**95%CI**
Fixed effects				
Constant	11.348 (0.055) ***	(11.239 to 11.457)	10.453 (0.136) ***	(10.187 to 10.720)
Random effects				
Level 3	0.058 (0.020) ***	(0.029 to 0.114)	0.360 (0.122) ***	(0.185 to 0.702)
Level 2	0.112 (0.006) ***	(0.101 to 0.125)	0.415 (0.024) ***	(0.371 to 0.463)
Residual	0.186 (0.001) ***	(0.186 to 0.188)	0.325 (0.001) ***	(0.324 to 0.327)
ICC				

Level 3	0.163 (0.047) ***	(0.090 to 0.276)	0.327 (0.075) ***	(0.199 to 0.487)
Level 2	0.477 (0.030) ***	(0.418 to 0.537)	0.704 (0.033) ***	(0.634 to 0.765)
Observation	383,664	383,664	383,664	383,664

Note. the summary statistics are calculated on the pre- matching sample. Significance level: *p < 0.05; **p < 0.01; ***p < 0.001.

Table S3. Multilevel linear analysis of factors associated with physicians' salary before matching

	**ln (Total annual salary)**		**ln (Annual performance-based salary)**	
	**Coeff. (SD)**	**95%CI**	**Coeff. (SD)**	95%CI
Fixed effects				
Group	0.034 (0.033) *	(-0.032 to 0.101)	0.169 (0.077) ***	(0.016 to 0.320)
ln (GDP per capita) (10 000 CNY)	-0.102 (0.148)	(-0.393 to 0.189)	0.562 (0.481)	(-0.380 to 1.506)
ln (average pay of the urban worker) (10 000 CNY)	1.36 (0.269) ***	(0.833 to 1.89)	1.429 (0.867) *	(-0.271 to 3.130)
Secondary hospital	0.072 (0.108)	(-0.138 to 0.283)	-0.149 (0.25)	(-0.643 to 0.344)
Tertiary hospital	0.293 (0.109) ***	(0.080 to 0.50)	0.107 (0.254)	(-0.391 to 0.605)
Traditional Chinese medicine hospital	-0.134 (0.028) ***	(-0.189 to -0.079)	-0.213 (0.062) ***	(-0.335 to -0.092)
Specialized hospital	-0.038 (0.045)	(-0.128 to 0.051)	-0.162 (0.100)	(-0.359 to 0.034)
Male	0.042 (0.001) ***	(0.039 to 0.045)	0.064 (0.00) ***	(0.060 to 0.069)
Age (years)	0.006 (0.001) ***	(0.005 to 0.006)	0.004 (0.000) ***	(0.004 to 0.005)
Establishment staff	0.170 (0.002) ***	(0.167 to 0.173)	0.115 (0.002) ***	(0.110 to 0.121)
Junior College	0.034 (0.002) ***	(0.029 to 0.038)	0.049 (0.003) ***	(0.042 to 0.056)
University	0.106 (0.002) ***	(0.101 to 0.110)	0.136 (0.00) ***	(0.128 to 0.143)
Masters and above	0.097 (0.003) ***	(0.091 to 0.103)	0.121 (0.004) ***	(0.112 to 0.131)
Primary professional title	0.350 (0.003) ***	(0.34 to 0.355)	0.444 (0.004) ***	(0.434 to 0.454)
Intermediate title	0.452 (0.003) ***	(0.445 to 0.458)	0.57 (0.005) ***	(0.563 to 0.585)
Associate senior title	0.554 (0.004) ***	(0.546 to 0.561)	0.658 (0.006) ***	(0.646 to 0.671)

Senior title	0.643 (0.005) ***	(0.633 to 0.652)	0.753 (0.007) ***	(0.738 to 0.769)
Section leader	0.137 (0.002) ***	(0.133 to 0.140)	0.182 (0.003) ***	(0.176 to 0.188)
Hospital manager	0.314 (0.007) ***	(0.29 to 0.328)	0.398 (0.012) ***	(0.375 to 0.422)
Medical detection departments	-0.030 (0.002) ***	(-0.033 to -0.027)	-0.040 (0.00) ***	(-0.045 to -0.035)
General Affairs/Logistics departments	-0.117 (0.008) ***	(-0.132 to -0.102)	-0.208 (0.012) ***	(-0.233 to -0.183)
Administrative departments	-0.136 (0.003) ***	(-0.141 to -0.130)	-0.194 (0.00) ***	(-0.204 to -0.185)
Other departments	-0.192 (0.003) ***	(-0.196 to -0.187)	-0.299 (0.004) ***	(-0.308 to -0.291)
Years of experience	0.006 (0.001) ***	(0.005 to 0.006)	0.003 (0.000) ***	(0.003 to 0.004)
Random effects				
Level 3	0.014 (0.006) ***	(0.006, 0.032)	0.177 (0.065) ***	(0.085, 0.365)
Level 2	0.081 (0.005) ***	(0.072, 0.092)	0.388 (0.025) ***	(0.342, 0.440)
Observation	383,664	383,664	383,664	383,664

Note. the summary statistics are calculated on the pre- matching sample. Significance level: *$p < 0.05$; **$p < 0.01$; ***$p < 0.001$.

Table S4. Subgroup analyses based on education level and gender

	Education status				Gender	
	Secondary vocational diploma/Junior high school	Vocational diploma	Bachelor	Masters and above	Female	Male
Fixed effects						
ln (Total annual pay)						

Group	0.05 (0.03) **	0.01 (0.03)	0.01 (0.03)	0.07 (0.04) *	0.02 (0.03)	0.01 (0.04)
ln (Performance-based pay)						
Group	0.19 (0.07) ***	0.14 (0.07) *	0.20 (0.07) **	0.21 (0.07) ***	0.19 (0.07) ***	0.169 (0.07) ***
Observation	7,854	52,808	98,306	19,792	139,855	38,766
